# Supplementary material for: Diagnostic performances of pepsinogens and gastrin-17 for atrophic gastritis and gastric cancer in Mongolian subjects
Source: PLoS One. 2022 Oct 17;17(10):e0274938. doi: 10.1371/journal.pone.0274938 (PMC9576095; doi:10.1371/journal.pone.0274938)
Supplement: Attachment — Submitted filename: Response to Rewiewers.docx [file pone.0274938.s001.docx]

**Manuscript ID: PONE-D-21-19578**

**Response to reviewers**

**Dear Editor**

Thank you for giving us the opportunity to submit a revised draft of the manuscript “***Diagnostic performances of serum Pepsinogens and Gastrin-17 for atrophic gastritis and gastric cancer in Mongolian subjects*”** for publication in the PLOS ONE. We appreciate the time and effort that you and reviewers dedicated to providing feedback on our manuscript and are grateful for the generous comments on and valuable improvements to our paper. Following reviewers’ suggestions, we have made a number of revisions to the manuscript. Those changes are highlighted within the manuscript. Changes to the manuscript are shown in blue.

We hope the revised version is now suitable for publication and look forward to hearing from you.

Here, we have answered each of the comments below.

Reviewer 2’s comments:

**Comment 1:** *Please provide a reference for the score used to combine the biomarkers with other risk factors.*

**Response:** There is no reference for the score which combines the biomarkers and risk factors. We designed it newly in order to improve diagnostic performance:

- For atrophic gastritis: giving one point to each of the age ≤40, PGI ≤75.07 ng/ml, PGR ≤6.25, and G-17 ≤23.42, a total of four scores
- For gastric cancer: giving one point to each of the age ≤40, positive family history of gastric cancer, positive previous disease history, PGI ≤35.25 ng/ml, and PGR ≤5.27, a total of five scores

We have added a comparison of our score and some results which are developed by other researchers in the Discussion section (Line 258-263).

**Comment 2:** *The sensitivity and specificity are relatively low, even after applying the score. I think this is not sufficient to reach this conclusion.*

**Response:** We agree with your comment that sensitivity and specificity are relatively low. Thus, we considered that these serum biomarkers and scores using serum biomarkers and risk factors could not identify gastric cancer directly. So we concluded PGI, PGR, and G-17 combined with the risk of age, family history, and previous gastric disease could identify individuals at high risk of gastric cancer and precancerous lesions who may need further examination, including upper endoscopy. According to your valuable comment, we have revised our conclusion. The revised conclusion reads as follows (Line 295-298):

[In conclusion, PGI, PGR biomarkers and combined with the risk of age, family history, and previous gastric disease could not be an alternative test of upper endoscopy, but might be a supportive method which is identifying individuals at high risk of gastric cancer and precancerous lesions who may need upper endoscopy]

Reviewer 3’s comments:

**Comment 1:** *The data plotted in Figure 1 demonstrate a very substantial overlap between the control population and the two disease populations, Atrophic Gastritis and Gastric Cancer. The graph of the PGR biomarker (pepsinogen ratios) shows a believable trend towards decrease with advancing disease. This is known from the literature. The data presented here and in Table 2, although showing a "significant" statistical difference in the populations, demonstrate that the serum assay cannot distinguish the benign from malignant disease. The data in both the figure and table demonstrate a very low clinical sensitivity for separation of the two disease populations from the healthy population.*

**Response:** We agree with this comment. Our result shows biomarkers could not distinguish the benign from malignant disease clearly and have relatively low sensitivity and specificity with the significant statistical differences in the study populations. Thus, we concluded biomarkers and biomarkers with risk factors could identify individuals at high risk of gastric cancer and precancerous disease who may need further examination. These blood tests may identify who needs an upper endoscopy. According to your comment, we have revised our conclusion (Line 295-298):

[In conclusion, PGI, PGR biomarkers and combined with the risk of age, family history, and previous gastric disease could not be an alternative test of upper endoscopy, but might be a supportive method which is identifying individuals at high risk of gastric cancer and precancerous lesions who may need upper endoscopy]

**Comment 2:** *The authors proposed a combined biomarker using 4 scoring factors. This is an interesting approach. However, not enough data was provided on the application of the 4 factors biomarker score on this study. A Table and Figure covering the population data set for all patients for the proposed marker should be included.*

**Response:** We enrolled 120 subjects, including 40 gastric cancer patients, 40 atrophic gastritis patients, and 40 healthy controls. After the exclusion of 6 subjects (positive history of *H.Pylori* eradication within three months and pregnancy) 36 with gastric cancer, 40 with chronic atrophic gastritis, and 38 healthy subjects were included in further analyses. All these subjects were included for the score. According to your comment, we reconstructed Table 2 and provided population data. Thank you for pointing out this.

**Comment 3:** *There is a Figure 2 in the manuscript, showing ROC plots for the two biomarkers selected for analysis, PGI (serum pepsinogen I) and PGR (serum pepsinogen I-II ratios). The plots appear to show a somewhat better performance of the markers when comparing gastric cancer to normal vs gastric atrophy to normal. The tabular analysis demonstrates that the serum marker levels will not distinguish the two diseases. Therefore the authors need to provide a cogent argument on how the serum assay would actually be useful in the clinic and what would be the benefit of a low sensitivity biomarker assay that cannot distinguish benign from malignant disease; the authors point out the lack of available endoscopy facilities in Mongolia but also point out that only pathology workup can distinguish benign from cancerous lesions; does this mean that endoscopy is unlikely to be useful or will endoscopy replace the serum assay in most circumstances due to better capability of separating normal from diseased (benign plus cancer) populations.*

**Response:** We suggest these biomarkers might be a supportive method for upper endoscopy. This idea was illustrated in the Discussion section: [Our finding indicated that these non-invasive biomarkers cannot be perfect alternative methods to endoscopic examination for the diagnosis of gastric cancer and precancerous lesions. However, these might be valuable screening markers for precancerous gastric lesions and gastric cancer] and [The use of endoscopic screening methods for gastric cancer has several limitations in our country, such as insufficient patient enrollment due to invasiveness, poor supply of endoscopic devices for all regions, and deficiency of well-trained endoscopists to meet the increased demand]. We revised some paragraphs in our Discussion to clarify our idea clearly. Thank you for your helpful comment.

**Comment 4:** *Line 80; solution is misspelled*

**Response:** Thank you for the correction. We have replaced “silution” with “solution” in the Materials and Method section (Line 80).

**Comment 5:** *Line 96; change wording to "following the manufacturer's package insert"*

**Response:** Thank you for your suggestion. We have changed "following the manufacturer's package insert" instead of “as instruction of kit” in the Materials and Method section (Line 95-96).

**Comment 6:** *Line 105;* *Automatically washed, this probably means with a plate washer; please specify including model used*

**Response:** Thank you for your suggestion. We used a BIOBASE-EL10A automated microplate washer and reader device (Biobase Biodustry, Shandong, China). We specify the washer model in the Materials and Method section, line 106.

**Comment 7:** *Lines 138-144; demonstrate that there is not an adequate rationale for including the PG-II and G17 measurements in the data analyses*

**Response:** Thank you for pointing out this. We demonstrated PGII and G-17 levels only in Table 1. According to your comment, we have added statements about PGII and G-17 levels of the study groups in the literature. The revised statement reads as follows:

[The median of PGII was 13.32 ng/ml (6.24 to 39.48) for healthy controls, 11.39 ng/ml (4.20 to 40.75) for atrophic gastritis and 16.60 ng/ml (3.73 to 41.77) for gastric cancer patients. And the median of G-17 was 13.99 pmol/l (0.80 to 78.89) for healthy controls, 11.39 pmol/l (0.96 to 45.80) for atrophic gastritis and 13.41 pmol/l (0.11 to 77.49) for gastric cancer patients. There were no significant differences in the serum PGII and G-17 levels between study groups (Fig 1B, 1D)] (Line 136-141).

**Comment 8:** *Line 162-164; given that there is no measurable difference across the populations for PG-II and G17, there inclusion to make the data more statistically significant is not justified*

**Response:** We apologize for the misunderstanding. PGII and G-17 have no significant difference between the study groups, as you said. Thus, we did not include these biomarkers in statistical analyses to determine diagnostic accuracy. We have made a typing error (PGI/II instead of PGR) in Table 2. That error may have caused you to misunderstand.

**Comment 9:** *Table 2; there are two columns labeled as positive likelihood ratio. Presumably one should be labeled negative; this reviewer does not understand what the authors mean by a positive likelihood ratio. Is this just that the biomarker is positive or is this a probability of disease. If the latter, is it restricted to cancer or is it either benign or cancer?*

**Response:** Thank you for your correction. I apologize for the typing error issue. We have corrected the column which represents the negative likelihood ratio (Table 2). We reconstructed the Table 2 to show outcome clearly.

**Comment 9:** *Line 225; the high background incidence of H pylori in Mongolia is a critical factor in understanding this study; this sentence should be moved to the introduction.*

**Response:** Thank you for your suggestion. According to your comment, we have moved statements about *H pylori* infection among the Mongolian population to the Introduction. The revised statement reads as follows (Line 30-32):

[Many epidemiological studies have revealed a strong association between *H. pylori* infection and gastric cancer. Previous studies have shown that the prevalence of *H. pylori* infection is high among the population of Mongolia]

**Comment 10:** *Lines 252-272; this paragraph is unnecessarily long and redundant, and should be reworked.*

**Response:** Thank you for your comment. We have reworked and minimized the paragraph (Line 271-287).

**Comment 11:** *Line 280 is unsupported by the data presented, and yet is likely the most important outcome of this study if true.*

**Response:** Line 293 [According to some studies, serum PGII and PGR levels are associated with *H. pylori* infection status and its eradication. (25, 27)]. That was not our outcome. We cited this outcome from Korean and Iranian studies. We have estimated biomarkers levels in *H. pylori* IgG negative and positive groups, and have added outcomes in our Result section (Line 156-165). And have added some discussion about our outcome in our Discussion section (Line 267-270). Thank you for your helpful comment.

Reviewer 4’s comments:

**Comment 1:** *The authors reported the H. pylori positive (58.8%) in the study groups but did not incorporate H. pylori results as a risk factor for chronic atrophic gastritis or gastric cancer. H. pylori IgG, age, gender, and smoking status have been reported as risk factors for gastric cancer. The authors did not cite either old or recent publications describing these four molecules for gastric cancer, for examples:*

*1) Cao et al. (2007) in Journal of Digestive Disease, “Screening of atrophic gastritis and gastric cancer by serum pepsinogen, gastrin-17 and Helicobacter pylori immunoglobulin G antibodies”, where H. pylori IgG results were also incorporated in the analyses with PGI and PGR. These authors reported the significant increase in G-17 in the gastric cancer group.*

*2) Reviewed publications, Kim N. & Jung C. (2010) Gut Liver “The role of serum pepsinogen in the detection of gastric cancer”.*

*3) Wang & Chen (2020) Scientific Reports, “Prevalence of atrophic gastritis in southwest China and predictive strength of serum gastrin-17: A cross-sectional study (SIGES)”*

*4) Shen et al. (2021) Gastroenterology Research and Practice “The diagnostic value of serum gastin-17 and pepsinogen for gastric cancer screening in eastern China”.*

**Response:** Thank you for your comment and suggestions of recent publications. Because *H. pylori* IgG has no significant difference between the study groups, we did not include to biomarker + risk factors score. According to your valuable suggestion, we have cited those publications and have added some statements in our Discussion section (Line 238-240, line 241-244, line 269).

**Comment 2:** *Line 80, “simethicone silution” should be “solution”.*

**Response:** Thank you for your correction. We have replaced “silution” with “solution” (Line 80).

**Comment 3:** *Line 83, the authors described the collection of biopsies, but nowhere in the manuscript described the results from these samples.*

**Response:** We agree with this comment. Thank you for pointing out this. We have added biopsy results of gastric cancer patients in the Result section (Line 129-132).

**Comment 4:** *Line 92 of the manuscript “The fasting blood samples were collected in an EDTA tube from all subjects.” The presence of EDTA as anticoagulant in the tube is for plasma collection, not serum, as the authors used “serum” throughout the manuscript.*

**Response:** We strongly agree with this comment. The pepsinogen I ELISA kit (Biohit, Helsinki, Finland) is a kit for the determination of human pepsinogen I from plasma or serum samples and blood samples are collected in EDTA or serum tube. We used an EDTA tube for sample collection. So, we made mistake in our literature. Thank you for your correction.

**Comment 5:** *Line 224, “In this study, H. pylori IgG was not associated with atrophic gastritis and gastric cancer”. Nowhere in the manuscript the authors described the analyses for PGI, PGR, and G-17 relative to H. pylori IgG positive and negative subgroups.*

**Response:** Our outcome shows that *H. pylori* IgG has no significant difference between gastric cancer patients, atrophic gastritis patients, and healthy controls. Thus, we did not include H. pylori status for further analyses. According to your comment, we have estimated PGI, PGII, PGR, and G-17 relative to *H. pylori* IgG positive and negative groups, and have added outcomes in our Result section. Our outcome shows that the PGI and PGII levels were higher in *H. pylori* positive subjects than in *H. pylori* negative subjects (p<0.01), while there was no difference in PGR and G-17 levels between them (Line 156-165). Thank you for your valuable comment.

In addition to the above comments, some spelling and grammatical errors pointed out by the reviewers have been corrected.

We look forward to hearing from you and responding to any further questions and comments you may have.

Sincerely,

Tulgaa Lonjid

February 15, 2022
