# Supplementary material for: Diagnostic performances of pepsinogens and gastrin-17 for atrophic gastritis and gastric cancer in Mongolian subjects
Source: PLoS One. 2022 Oct 17;17(10):e0274938. doi: 10.1371/journal.pone.0274938 (PMC9576095; doi:10.1371/journal.pone.0274938)
Supplement: Attachment — Submitted filename: Response to Rewiewers_second.docx [file pone.0274938.s002.docx]

**Manuscript ID: PONE-D-21-19578**

**Response to reviewers**

**Dear Editor**

Thank you for giving us the opportunity to submit a revised draft of the manuscript “***Diagnostic performances of serum Pepsinogens and Gastrin-17 for atrophic gastritis and gastric cancer in Mongolian subjects*”** for publication in the PLOS ONE. We appreciate the time and effort that you and reviewers dedicated to providing feedback on our manuscript and are grateful for the generous comments on and valuable improvements to our paper. Following reviewer’s suggestions, we have made a number of revisions to the manuscript. Those changes are highlighted within the manuscript. Changes to the manuscript are shown in blue.

We hope the revised version is now suitable for publication and look forward to hearing from you.

Here, we have answered each of the comments below.

Reviewer 2’s comments:

## Comment 1: *Please provide a reference for similar scoring. Why the score variables are different regarding AG and GC? The score used need to be clearly mentioned in the methods section.* Response: We strongly agree with your comment that score variables are different. So, we conducted new analysis and created new scoring system combined with atrophic gastritis and gastric cancer. We have added our results under additional subtitle “Scoring system to predict risk of atrophic gastritis and gastric cancer” (Line 206-222). Cai Q et al (2019) comprised seven variables, including age, sex, PGR, G-17 level, *H. pylori* infection, pickled food and fried food, with scores from 0 to 25 to stratify high-risk population in China (ref 24). Thank you for your valuable

**Comment 2:** *The score used need to be clearly mentioned in the methods section.*

**Response:** According to your comment, we have mentioned about the scoring system in the method section (Line 120-124). Thank you for pointing out this.

We look forward to hearing from you and responding to any further questions and comments you may have.

Sincerely,

Tulgaa Lonjid

July 8, 2022
