# Supplementary material for: Diagnostic performances of pepsinogens and gastrin-17 for atrophic gastritis and gastric cancer in Mongolian subjects
Source: PLoS One. 2022 Oct 17;17(10):e0274938. doi: 10.1371/journal.pone.0274938 (PMC9576095; doi:10.1371/journal.pone.0274938)
Supplement: Attachment — Submitted filename: Response to Reviewers.docx [file pone.0274938.s004.docx]

**Manuscript ID: PONE-D-21-19578**

**Response to reviewer**

**Dear Editor**

Thank you for giving us the opportunity to submit a revised draft of the manuscript “***Diagnostic performances of serum Pepsinogens and Gastrin-17 for atrophic gastritis and gastric cancer in Mongolian subjects*”** for publication in the PLOS ONE. We appreciate the time and effort that you and reviewers dedicated to providing feedback on our manuscript and are grateful for the generous comments on and valuable improvements to our paper. Following reviewer’s suggestion, we have made a minor revisions to the manuscript. Those changes are highlighted within the manuscript. Changes to the manuscript are shown in blue.

We hope the revised version is now suitable for publication and look forward to hearing from you.

Here, we have answered comment below.

Reviewer 2’s comments:

## Comment: *The authors didn't provide a reference for the used score. Have the authors proposed this score? If so, then the conclusion needs to be modified as they are not evaluating a well established score.*

Response: We strongly agree with your comment. We have added reference score of medium- and high risk in round bracket to make it more clear (Line 274-276). Also, we have modified our conclusion according to your comment that conclusion needs to be proposed scoring system (Line 309-313). We considered that our scoring system could identify individuals who may need upper endoscopy. The use of endoscopic screening method for gastric cancer has several limitations in our country, such as insufficient patient enrollment due to invasiveness, poor supply of endoscopic devices for all regions, and deficiency well-trained endoscopist to meet the increased demand. Therefore, these biomarkers, combined with the risk of age, family history, and previous gastric disease might be considered supportive method for the mass screening of gastric cancer and precancerous lesions in our country. Thank you for your useful comments. We believe that our manuscript are improving with your valuable suggestions.

We look forward to hearing from you and responding to any further questions and comments you may have.

Sincerely,

Tulgaa Lonjid

August 30, 2022
